# Transplant nephrectomy: indication, surgical approach and complications—experiences from a single transplantation center

**DOI:** 10.1007/s00345-024-04884-8

**Published:** 2024-03-06

**Authors:** Josef Mang, Josephine Haag, Lutz Liefeldt, Klemens Budde, Robert Peters, Sebastian L. Hofbauer, Matthias Schulz, Sarah Weinberger, Julia Dagnæs-Hansen, Andreas Maxeiner, Bernhard Ralla, Frank Friedersdorff

**Affiliations:** 1 Department of Urology, Charité–Universitätsmedizin Berlin, Corporate Member of Freie Universität Berlin, Humboldt-Universität Zu Berlin, and Berlin Institute of Health, Charitéplatz 1, 10117 Berlin, Germany; 2 Department of Nephrology, Charité–Universitätsmedizin Berlin, Corporate Member of Freie Universität Berlin, Humboldt-Universität Zu Berlin, and Berlin Institute of Health, Berlin, Germany; 3 Urologic Research Unit, Department of Urology, Copenhagen University Hospital, Copenhagen, Denmark; 4 Department of Urology, Evangelisches Krankenhaus Königin Elisabeth Herzberge, Berlin, Germany

**Keywords:** Failure kidney allograft, Transplant nephrectomy, Indications for transplant nephrectomy, Intracapsular, Extracapsular, Complications

## Abstract

**Purpose:**

Management of a failed kidney allograft, and the question whether it should be removed is a challenging task for clinicians. The reported risks for transplant nephrectomy (TN) vary, and there is no clear recommendation on indications or surgical approach that should be used. This study gives an overview of indications, compares surgical techniques, and identifies risk factors for higher morbidity.

**Methods:**

Retrospective analysis was conducted on all transplant nephrectomies performed between 2005 and 2020 at Charité Hospital Berlin, Department of Urology. Patient demographics, laboratory parameters, graft survival data, indication for TN, and surgical complications were extracted from medical reports.

**Results:**

A total of 195 TN were performed, with graft intolerance syndrome being the most common indication in 52 patients (26.7%), acute rejection in 36 (18.5%), acute infection in 30 (15.4%), and other reasons to stop immunosuppression in 26 patients (13.3%). Rare indications were vascular complications in 16 (8.2%) and malignancies in the allograft in six (3.1%) cases. Extracapsular surgical approach was significantly more often used in cases of vascular complications and earlier allograft removal, but there was no difference in complication rates between extra- and intracapsular approach. Acute infection was identified as an independent risk factor for a complication grade IIIb or higher according to Clavien–Dindo classification, with a HR of 12.3 (CI 2.2–67.7; *p* = 0.004).

**Conclusion:**

Transplant nephrectomy should only be performed when there is a good indication, and non-elective surgery should be avoided, when possible, as it increases morbidity.

## Introduction

Kidney transplantation is the therapy of choice for end stage renal disease and, therefore, numbers of patients who received a graft have been rising [1]. Transplant failure (TF) in the first year is about 9–11%, increasing to about 23–38% after 5 years and 54–66% after 10 years [2]. After kidney TF surgical removal of the allograft has to be evaluated. Whereas there are rarely debated indications for transplant nephrectomy (TN), such as arterial or venous thrombosis, acute rejection or infection of the allograft, indications for TN in chronic transplant failure are not as clearly defined [3], resulting in TN rates between 4.5 and 84.4% depending on centers policy [4]. Possible positive effects of retaining the transplant, such as erythropoetin (EPO) production, residual kidney function, or avoiding risks for morbidity and mortality through the operative procedure, have to be weighed against retaining a nonfunctional allograft as a cause for chronic inflammation or development of a graft intolerance syndrome (GIS) presenting with allograft tenderness, fever, and hematuria [4, 5].

Besides the operative risks, TN is considered as an event of immunization, causing higher levels of panel reacting antibodies (PRA) in patients who had undergone TN, compared to patients who retained their allograft, resulting in a higher risk for delayed graft function or primary non-function after re-transplantation [6–8].

Once decision for TN is made, there are two surgical approaches: an extracapsular approach where the complete allograft is removed with its capsule, compared to an intracapsular approach where the plane between parenchyma and capsule is developed to excise the kidney [9]. However, combinations of both techniques are often used depending on intraoperative findings and possibility to develop the different planes.

After TF, considering all these factors for decision-making can be a challenging task for clinicians and, therefore, indication and timing of the procedure must be evaluated carefully. This study aims to show TN results of a single transplant center, regarding indication, complications, surgical approach, and TN outcome.

## Materials and methods

### Patients and parameters

For this study, all adult patients who underwent TN between January 2005 and February 2020 at Charité Hospital Berlin, Department of Urology, were analyzed retrospectively. Patient demographics, laboratory parameters and graft survival data including clinical information on indication for TN and surgical complications were extracted from their medical reports. This study was performed according to the ethical approval of the institutional review board.

### Surgical technique

TN was performed either by extracapsular or intracapsular approach depending on anatomical conditions, time after transplantation and surgeons’ preferences. For intracapsular TN, the capsule was incised, and the transplant was dissected within the capsule, whereas the ureter and capsule remained in situ. For extracapsular approach, the transplant was removed with the capsule. In cases with combinations of both surgical techniques, the determination to either method for further evaluation in this study, was made based on the predominantly used technique according to the surgeon’s report. Drain and perioperative antibiotic prophylaxis were used routinely. To acquire the presented data, all medical reports and operation protocols were studied and postoperative complications were evaluated regarding the Clavien–Dindo classification for surgical complications [10].

### Statistics

All statistical calculations were performed using IBM® SPSS® Statistics Version 22 (IBM Inc., Armonk, New York). Fisher´s exact test was used for nominal parameters and Mann–Whitney *U* test for nonparametric tests for ordinal or continuous parameters. Logistic regression models were used for multivariate analysis. A p value of less than 0.05 was considered statistically significant.

## Results

TN was performed in 195 patients, comprising of 119 (61%) men and 76 (39%) women with a median age of 51.4 years and a median transplant survival of 64 months. Patient characteristics are presented in Table 1. In 117 patients (60%), intracapsular surgical approach was used, whereas extracapsular technique was used in 36 patients (18.5%). Complications were detected in 99 patients (50.8%), of which 25 (12.8%) needed intervention under general anesthesia.

**Table 1:** Patient characteristics

Characteristic	*N* = 195 (%)
Sex	
Male	119 (61)
Female	76 (39)
Surgical technique	
Intracapsular	117 (60)
Extracapsular	36 (18.5)
All postoperative complications	
Yes	99 (50.8)
No	91 (46.7)
Postoperative complication > 3b	
Yes	25 (12.8)
No	165 (84.6)
Multiple arteries/veins	
Yes	41 (21)
No	111 (56.9)
Prior transplantation	
Yes	48 (24.6)
No	147 (75.4)
	Mean (range)
Age [years]	51.4 (15–79)
Transplant survival [months]	64 (0–376)
Time of surgery [min]	138 (55–433)
Hospital stay [days]	11 (3–140)

Looking on indications (Table 2) for TN in our cohort, graft intolerance syndrome most often was the underlying cause for TN in 52 patients (26.7%), followed by acute rejection in 36 (18.5%) cases, acute infection in 30 (15.4%) patients and other reasons to stop immunosuppression in 26 (13.3%) patients. More rare indications were vascular complications in 16 patients (8.2%) or malignancies in the allograft in 6 (3.1%) cases. Also, 21 (10.7) patients underwent TN for other reasons. In eight patients (4.1%), the indication for TN retrospectively could not be determined.

**Table 2:** Indication for transplant nephrectomy

Indication	*N* = 195 (%)
Graft intolerance syndrome	52 (26.7)
Acute rejection	36 (18.5)
Acute infection	30 (15.4)
To stop immunosuppression	26 (13.3)
Vascular complication	16 (8.2)
Malignancy in the allograft	6 (3.1)
Other reasons	21 (10.7)
Unknown	8 (4.1)

Next, we wanted to look for possible differences depending on whether intracapsular or extracapsular surgical approach was used. Descriptive statistics on all parameters examined are shown in Table 3. Significant differences were found when indication for TN was an acute vascular complication. Here, significantly more often an extracapsular surgical approach was used in 11 (30.6%) patients compared to 3 (2.6%) patients with intracapsular surgical approach (*p* > 0.001). In line with this finding, an extracapsular surgical approach was used significantly more often in earlier transplant loss, than intracapsular surgery (50 months vs. 67 months, respectively, *p* = 0.003). Interestingly, we found a difference in the rate of complications grade IIIb or higher according to Clavien–Dindo classification for surgical complications [10], indicating a higher complication rate in extracapsular 8 (22.9%) compared to intracapsular 10 (8.5%) surgical approach (*p* = 0.034%).

**Table 3:** Intra- vs extracapsular surgical approach

Parameter	Intracapsular	Extracapsular	p-value
Indication (percent)			
Graft intolerance syndrome	35 (29.9)	5 (13.9)	0.081
Acute infection	20 (17.1)	6 (16.7)	1.0
Acute rejection	21 (17.9)	3 (8.3)	0.199
To stop immunosuppression	18 (15.4)	4 (11.1)	0.6
Vascular complications	3 (2.6)	11 (30.6)	< 0.001
Malignancy of the graft	4 (3.4)	0 (0)	0.573
Other reasons	11 (9.4)	6 (5.1)	0.234
Complications (percent)			
Any complication	55 (47)	21 (60)	0.248
Complication > = IIIb	10 (8.5)	8 (22.9)	0.034
Blood transfusion	23 (19.7)	8 (22.9)	0.643
Median age at TN [y]	55	47	n.s
Median time between TF and TN [d]	209	105	n.s
Median graft survival [m]	67	50	0.003
Median time of surgery [min]	138	140	n.s
Median stay at hospital [d]	10	13	n.s

*TN* transplant nephrectomy

*TF* transplant failure

In our collective 25 complications grade IIIb or higher occurred, consisting of bleeding complications in 8 cases, wound dehiscence in 3 cases, urinoma in 2 cases, thrombosis of the renal vein, sigma perforation and dialysis shunt thrombosis. In nine cases of grade IV and V complications, one patient suffered from myocardial infarction, all other eight patients form septic multiple organ dysfunctions. To further investigate these findings, we looked for other factors contributing to a higher complication rate. Thereby, we found that the indication of acute infection had significant more complications than other indications (44.8% vs. 7.5%, *p* > 0.0001). Next, we aimed to determine the factor each parameter contributed to a higher complication rate and therefore performed a multivariate logistic regression analysis, examining the parameters already identified and other possible factors as shown in Table 4. Here, the initial descriptive finding of the surgical approach as a risk factor for a higher complication rate could not be confirmed. However, acute infection as an indication for TN could be confirmed as one independent risk factor. With a hazard ratio (HR) of 12.3 (CI 2.2–67.7; *p* = 0.004), it seems to have a strong impact on the risk to suffer a higher grade complication.

**Table 4:** multivariate regression analysis for complications grade IIIb or higher

Variable	HR	95% CI	p-value
Indication—acute infection	12.269	2.22–67.74	0.004
Surgical technique	1.936	0.41–9.16	0.405
Patient age	0.976	0.92–1	0.382
Transplant surival	0.995	0.98–1	0.602
Time between TF and TN	0.994	0.99–1	0.142
Multiple vessels	2.189	0.54–8.87	0.272

*TN* transplant nephrectomy, *TF* transplant failure, *HR* hazard ratio, *CI* confidence intervals

## Discussion

When it comes to deciding whether a non-functional kidney allograft should be removed, opinions and recommendations vary, since there is currently no prospective data on (a) risks for the patients from surgery itself, and (b) implications for future re-transplantation. The retrospectively analyzed cohort in this work, with 195 included patients, is one of the largest single center cohorts. Although the biggest limitation of this study is clearly its retrospective design, it adds value to the discussion since risks for complications and risk factors for those become clearer for everyday decision making. In previous publications risks for complications due to TN vary widely with rates as high as 80% [11]. However, most of those studies have rather small patient cohorts, and larger and more recent studies report severe complications (grade IIIb or higher) at around 10% [11, 12] –16% [13]. The present study shows overall complication rates of 50.8% and 12.8% for complications grade IIIb or higher according to Clavien–Dindo classification. Those results are in line with other published larger cohorts of TN [11–13].

Furthermore, we found that TN performed due to acute infection was an independent risk factor with a HR of 12.3. This finding confirms the observation that urgent TN, especially in septic patients, leads to higher morbidity, as reported by Secin et al. and others in, however, smaller patient cohorts [14, 15].

Besides the perioperative risk for the patients due the procedure itself, possible immunological effects with implications for further transplantations must be considered, and although this study did not investigate this subject, it plays an important role for the indication for surgery. A larger meta-analysis from 2021 investigated the impact of TN on immunization and re-transplantation. They found higher levels of PRA, higher risk for delayed graft function and primary non-function. However, this did not translate into worse 5-year graft survival and patient survival [8].

Taken together those risks, our approach for TN is rather strict and we do not perform TN without real indication. In the present study, we identified indications that are similar to most published recommendations for TN [13, 16]. However, since there still are advocates to remove every failed graft, we eagerly await the results of the first prospective multicenter study, comparing systematic TN with conventional care after kidney graft failure (DESYRE) [17].

Considering the higher risk for perioperative complications in non-elective circumstances, such as acute infection or acute rejection, if possible, stabilization with subsequent surgery might be a better option. To address this, we have started treating patients with cortisone pulse therapy (500 mg prednisolone) in case of acute rejection or calculated broad spectrum antibiotics in case of acute infections instead of emergency surgery whenever clinically justifiable. Whether these actions will reduce perioperative risks remain to be evaluated. In an elective setting, however, for patients suffering mainly from GIS, another treatment option might be percutaneous embolization of the failed allograft, that can be performed under local anesthesia. A recent meta-analysis showed lower morbidity compared to TN, but 20% of patients needed a post-embolization nephrectomy none the less [18]. Considering other surgical techniques, there are few small case series of laparoscopic or robotic assisted TN [19–21]. With heterogenous results on operating time, mean hospital stay is about 4 days and no major complications are reported yet [19, 21]. However, so far, this technique has only been used in highly selected patients and there are no studies comparing laparoscopic to open techniques. Further larger studies are needed to answer whether this approach will be a better option than open surgery.

## Data Availability

The data that support the findings of this study are available on request from the corresponding author.

## Abbreviation

CI: Confidence interval
EPO: Erythropoetin
GIS: Graft intolerance syndrome
HR: Hazard ratio
PRA: Panel reacting antibodies
TF: Transplant failure
TN: Transplant nephrectomy
